# *Drosophila melanogaster* Y Chromosome Genes Affect Male Sensitivity to Microbial Infections

**DOI:** 10.3390/insects12010030

**Published:** 2021-01-05

**Authors:** Gloria Bartolo, Leandra O. Gonzalez, Anastasia Levitin, Mikhail Martchenko Shilman

**Affiliations:** Henry E. Riggs School of Applied Life Sciences, Keck Graduate Institute, 535 Watson Drive, Claremont, CA 91711, USA; gbartolo18@students.kgi.edu (G.B.); lgonzalez19@students.kgi.edu (L.O.G.); anastasia_levitin@kgi.edu (A.L.)

**Keywords:** *Drosophila melanogaster*, *Saccharomyces cerevisiae*, *Serratia liquefaciens*, microbial infections, Y chromosome, sexual dimorphism

## Abstract

**Simple Summary:**

This manuscript focuses on the protein-encoding genes of the *Drosophila melanogaster* Y chromosome and their role in immunity. Since their discovery, these genes have mainly been studied for their role(s) in male fertility, such as spermatogenesis. Two previously published papers have linked the polymorphism within the Y chromosome and immunity. Currently, there is no research article to our knowledge that has studied the effect of the individual genes of the Y chromosome on fly immunity. Here, we aim to address the lack of immunity-related knowledge of Y chromosome genes by determining the effect of many of these genes on male sensitivity to microbial infections. We challenged loss-of-function gene mutants with bacterial and fungal pathogens orally and observed any significant fly survival changes. We discovered several genes affecting male sensitivity to both bacterial and fungal infections. While most of the Y chromosome genes were found to reduce sensitivity, we found one gene increases sensitivity to fungal infection. Because several genes were found to increase male sensitivity to microbial infections, inhibitors for such genes could be introduced in areas where fruit flies are pests. Amid declining bee populations, Diptera pollinators could be protected from fungal infections with Y-gene inhibitors.

**Abstract:**

The genders of *Drosophila melanogaster* vary in their sensitivities to microbial pathogens. While many of the immunity-related genes are located on the X chromosome, the polymorphisms within the Y chromosome were also shown to affect the immunity of flies. In this study, we investigated the necessity of individual genes on the Y chromosome (Y-genes) for male sensitivity to microbes. We identified several Y-genes whose genetic inactivation either increases or decreases the sensitivity of males to gastrointestinal infections with fungal *Saccharomyces cerevisiae* and bacterial *Serratia liquefaciens*. Specifically, the loss of function mutations in fly kl-5 and Ppr-Y Y-genes lead to increased and decreased sensitivity of males to fungal challenge, respectively, compared to female sensitivity. In contrast, mutations in *Drosophila* Pp1-Y1, kl-5, kl-3, Ppr-Y, CCY, and FDY Y-genes lead to increased sensitivity of males to bacterial infection, compared to females. Moreover, while these Y-genes are necessary, the Y chromosome is not sufficient for the sensitivity of males to microbes, since the sensitivity of XXY females to fungal and bacterial challenges was not different from the sensitivity of wild-type female flies, compared to males. This study assigns a new immunity-related function to numerous Y-genes in *D.* *melanogaster*.

## 1. Introduction

When combatting pathogenic infections, *Drosophila* exhibit sexual dimorphism in innate immune responses and survival [1]. Innate immunity in *Drosophila melanogaster* is primarily regulated by antimicrobial peptides (AMPs) located on the second and third chromosomes [2,3]. AMP synthesis and secretion is controlled by two NF-κB-activating pathways: Imd and Toll [4]. While both pathways function to combat all microbial pathogens and their virulence factors, each pathway primarily fights against specific pathogens. The Imd pathway is primarily induced by Gram-negative bacteria, while the Toll pathway is triggered by the detection of Gram-positive bacteria and fungi. In addition to AMPs, macrophage-like plasmatocytes phagocytize microbial invaders immediately upon infection [4]. Certain pathogenic virulence factors also induce specific mechanisms within *Drosophila*. The pore-forming toxin of *Serratia marcescens* causes thinning of gut epithelium via purging of enterocyte cytoplasm, protecting enterocytes by extruding damaged cell components and invading bacteria and/or toxins [5].

Several *Drosophila* immunity related-genes are located on the X chromosome, including those critical for microbial recognition and signal transduction within Imd and Toll pathways [6]. The Toll pathway is mostly activated by lysine-type peptidoglycan found in most Gram-positive bacterial cell walls and by yeast cell wall components. In contrast, the Imd pathway is primarily activated in response to diaminopimelate-type peptidoglycans found in all Gram-negative bacteria and Gram-positive *Bacillus* species. Due to their location in a sex chromosome, the expression of such immunity genes within male and female flies is important, as variance in expression may contribute to the sexual dimorphism in immunity seen during pathogenic infections. Unlike mammals, where females randomly silence one of their X chromosomes to maintain a balance between sexes, *Drosophila* males increase their single X chromosome expression two-fold [7,8]. Compensation of X chromosome, and subsequently X-linked immunity-related genes, is important as genetic variation within natural populations can expose flies to alleles that act in a sex-specific manner, resulting in sex differences in immune competence [6].

In addition to the X chromosome, the Y chromosome may also contribute to differences in the sensitivity of genders to pathogens. Although essential for male fertility in most *Drosophila* species [9,10], the Y chromosome lacks sex determination genes [11]. Instead, sex determination relies on X chromosome dosage: X0 and XY flies are male, and XX and XXY flies are females [11,12]. The Y chromosome of *D. melanogaster* is comprised of the following genes (Y genes): Pp1-Y1, kl-5, PRY, kl-3, kl-2, ARY, Ppr-Y, WDY, FDY, Mst77y (multiple gene copies [13]), Pp1-Y2, ORY, and CCY. Multiple Y- genes (kl-5, PRY, kl-3, kl-2, WDY, ORY, and CCY) are essential and may contribute to fertility [10,14,15]. Although not directly immune-related, the Y chromosome may play a role in the sexual dimorphism of innate immunity. Only the addition of the polymorphic non-transcribed Y chromosomes to isogenic females alters the relative expression of AMPs [16]. Varying Y chromosomes within populations may also alter AMP gene expression, altering flies’ ability to combat bacterial infection [17].

## 2. Materials and Methods

### 2.1. Drosophila Rearing

*Drosophila melanogaster* strains were housed at 25 °C with 12 h light/dark cycles and fed on standard cornmeal–molasses–agar fly medium with yeast flakes. Wild-type experiments were conducted with Oregon-R, selected for their rapid egg-laying ability (Bloomington *Drosophila* Stock Center (BDSC) stock #2376), *Drosophila* aged 4–5 days. Experiments with Y-gene loss-of-function *Drosophila* utilized unaged flies at the time of the experiments. Mutants of Y-genes (BDSC #) include: ABO-X2 (XXY females) (790), CCY (61959), FDY (62536), kl-3 (53317), kl-5 (2763), Pp1-Y1 (58098), Pp1-Y2 (57236), Ppr-Y (33882), PRY (58235), and WDY (63650). Mutants of kl-5 were generated by x-ray mutagenesis. For mutants CCY, FDY, Pp1-Y1, Pp1-Y2, and PRY, homozygotes of RNAi targeting the gene of interest were chosen for survival assays. Heterozygotes were chosen for WDY as homozygotes were not present in abundance. For mutants Ppr-Y and kl-3, both heterozygotes and homozygotes were chosen. All ABO-X2 flies are homozygous mutants.

### 2.2. Drosophila Oral Feeding Survival Assay

*Saccharomyces cerevisiae* diploid strain YEF473, ATCC^®^ 200,970 [18], and *Serratia liquefaciens* (ATCC 27592) were used as the infective agent for all *Drosophila* survival assays. *S. cerevisiae* was incubated on yeast extract-peptone-dextrose (YPD) medium and *S. liquefaciens* incubated on Tryptic Soy Broth (TSB), both at 30 °C. Overnight cultures were grown in YPD or TSB at 30 °C at 180 rpm for 14–16 h. Flies were infected according to the microbial intestinal infection methods described previously in Nehme, et al. [19] with the following modifications. *Drosophila* vials were prepared by placing three 25 mm diameter circles of extra-thick Whatman blotting paper (Bio-Rad Laboratories, catalog #1703965) at the bottom of the vials and capping with a foam plug. *S. cerevisiae* or *S. liquefaciens* overnight cultures were centrifuged, and the pellets resuspended in 50mM (10%) sucrose solution to a final desired optical density at 600 nm (OD600) of 1.7 OD or 4.6 OD for *S. cerevisiae* and *S. liquefaciens*, respectively. OD600 values were converted to cells/mL: for yeast, OD600 of 1.0 corresponds to approximately 107 cells/mL [20]; for bacteria, OD600 values were converted according to McFarland’s scale [21]. Each prepared *Drosophila* vial contained 2.5 mL of its respective solution (absorbed by the Whatman paper found at the bottom of the vial). Flies were anesthetized by CO2, separated by gender, and placed into the *Drosophila* vials with ten flies in each vial. Vials were incubated at 30 °C and checked a minimum of twice per day for fly survival.

## 3. Results

### 3.1. Y Genes Repress or Contribute to the Sensitivity of Fly Males to Yeast Infection

**Figure 1:**
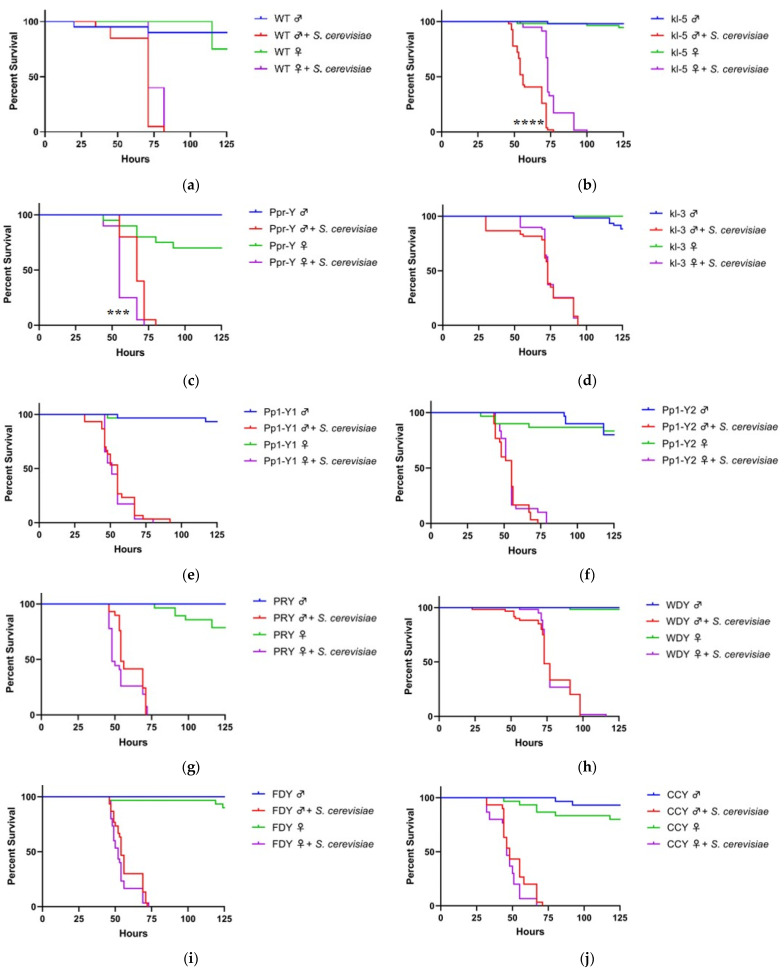

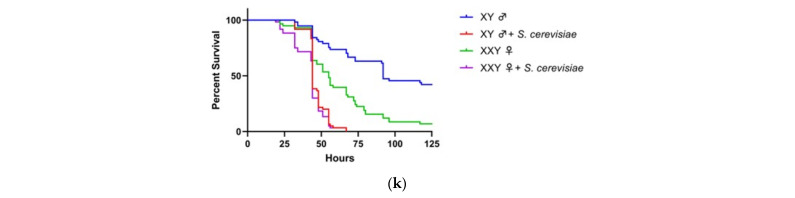
Effects of loss of function of Y-genes or gain of Y chromosome on the sensitivity of fly survival to yeast infection. Wild-type (WT), loss of function Y-mutant males and XXY females (ABO-X2) were orally exposed to a 50 mM sucrose solution containing *Saccharomyces cerevisiae*. For experiments with wild-type flies, Oregon-R strain was used (**a**). For mutant fly strain experiments involving loss of function of Y-genes, females are wild-type (**b**–**j**). In the case of XXY females, males are wild-type (**k**). Each condition contains ten flies. Vials are incubated at 30 °C and checked a minimum of twice per day for fly survival (***, *p* < 0.001; ****, *p* < 0.0001).

Sex biases of survival to infections in flies vary from pathogen to pathogen: fungal infection via *Candida albicans* favors female survival [22], while *Beauveria bassiana* favors male survival [16,23]. Survival can also depend on the mode of infection, as systemic immunity is driven by the production of AMPs, and intestinal immunity is driven by AMPs and reactive oxygen species [4]. We investigated the male and female sensitivity to microorganisms administered orally, as flies are naturally exposed to pathogens when looking for food. Although a commensal microorganism [24], *Saccharomyces cerevisiae* is lethal to wild-type flies during continuous oral infection. We observed that males and females are equally sensitive to yeast infection, with a median survival of 71 h for both genders (Figure 1a).

We investigated whether loss of function of Y-genes changes inter-gender sensitivities to *S. cerevisiae*. Genes omitted in this study include kl-2, ARY, Mst77y, and ORY because these mutant flies could not be propagated. In all the mutant strains, females were wild-type for these genes. The loss of function of kl-5 and Ppr-Y significantly altered gender survival bias during *S. cerevisiae* infection. Male flies with the mutation in kl-5 became more sensitive to yeast compared to females, resulting in median survival of 56 h, in comparison to a median survival of 73 h of kl-5 mutant females (Figure 1b). Conversely, male Ppr-Y mutants were less sensitive to yeast than female flies, with a median survival of 67 and 55 h, respectively (Figure 1c). No difference in survival sex bias was observed in the other seven gene mutants (Figure 1d–j).

ABO-X^2^ mutation is known to result in females with XXY genotype [25]. We investigated whether the addition of the Y chromosome to XX females is sufficient to affect the survival bias of genders during *S. cerevisiae* infection. In the absence of the infection, XXY females’ survival time is significantly less than their male counterparts (37 h difference in median survival; *p* < 0.0001) (Figure 1K). Notwithstanding the difference in gender longevity, we observed no difference in the survival between XXY female flies and XY male flies, suggesting the Y chromosome is not sufficient to affect the sex bias in survival during *S. cerevisiae* infection.

### 3.2. Y Genes Repress the Sensitivity of Fly Males to Bacterial Infection

**Figure 2:**
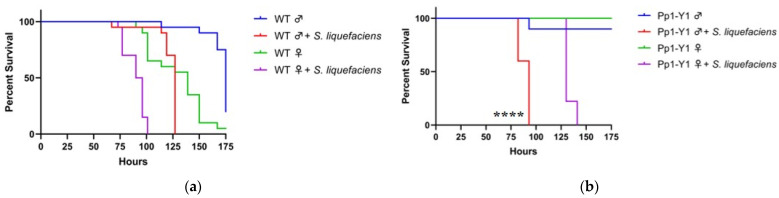

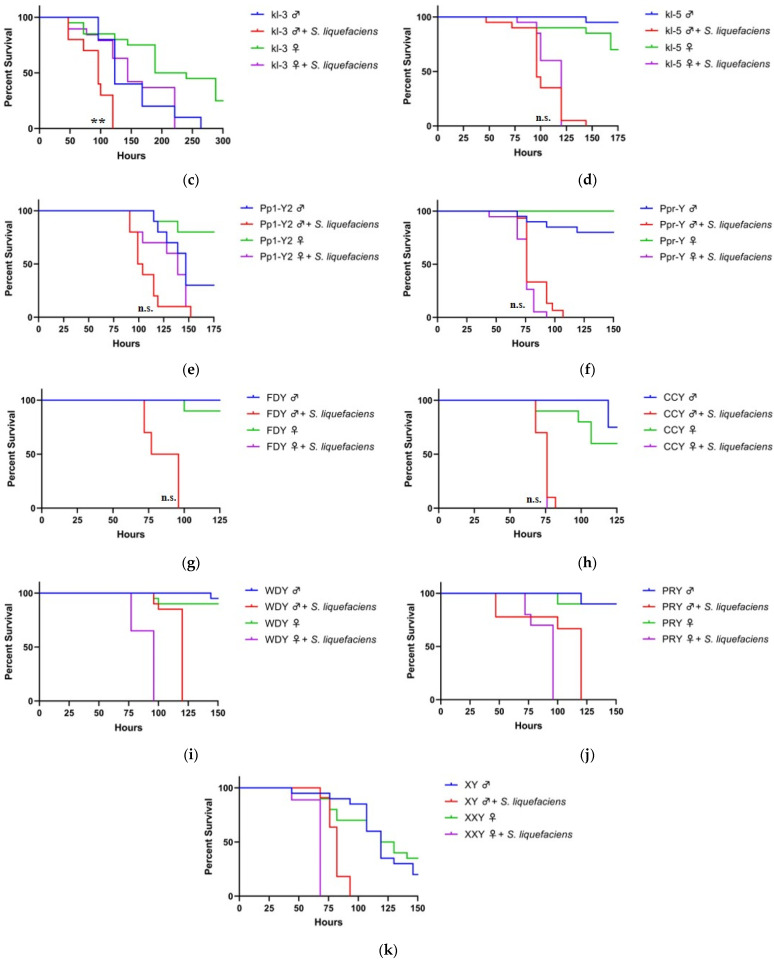
Loss of function of multiple Y-genes increase the sensitivity of males to bacterial infection. Wild-type (WT), loss of function Y-mutant males and XXY females (ABO-X2) were orally exposed to a 50 mM sucrose solution containing *Serratia liquefaciens.* For experiments with wild-type flies, Oregon-R strain was used (**a**). For mutant fly strain experiments involving loss of function of Y-genes, females are wild-type (**b**–**j**). In the case of XXY females, males are wild-type (**k**). Each condition contains ten flies. Vials are incubated at 30 °C and checked a minimum of twice per day for fly survival (**, *p* < 0.01; ****, *p* < 0.0001).

Although systemic innate immune responses in *Drosophila* are primarily regulated by Imd and Toll pathways, the Imd pathway regulates the gut immune response in the midgut, the primary site of digestion and absorption [4]. Additionally, while *S. cerevisiae* is recognized by Toll pathway, the Imd pathway primarily recognizes Gram-negative bacteria. Unlike *S. cerevisiae* infection, there is a sex bias during oral infection of *Serratia liquefaciens*, a Gram-negative bacterium. Wild-type male flies exhibit greater overall survival, with a median survival of 127 h, than the median survival of 93 h for females (Figure 2a).

Loss of function of several Y-genes altered the survival bias of male and female flies during *S. liquefaciens* infection. Specifically, mutations in Pp1-Y1 and kl-3 resulted in males becoming more sensitive than their respective females. Male Pp1-Y1 mutant flies exhibit a significantly shorter median survival time of 93 h than the median female survival of 130 h (Figure 2b). A similar trend is seen in kl-3 mutant flies, with median survival times of 96 and 144 h for males and females, respectively (Figure 2c). Additional Y-genes also affecting male sensitivity were kl-5, Pp1-Y2, Ppr-Y, CCY, and FDY. The loss of function in these genes leads to an increased male sensitivity such that no significant gender difference in median survival was observed (Figure 2d–g).

In contrast, WDY and PRY appear to not be immunity-related during oral *S. liquefaciens* infection, as the loss of function in these genes did not affect male sensitivity compared to females: similar to wild-type, the median male survival is 20 h greater than median female survival (Figure 2h–i). Additionally, ABO-X^2^ XXY females also have reduced median survival compared to males, suggesting the presence of the Y chromosome is not sufficient to affect the sex bias in survival during *S. liquefaciens* infection (Figure 2j).

## 4. Discussion

Here, we observe several Y-genes to have immunity-related functions. Very little is known about the function of Y-genes, with most functions described as fertility-related [23]. All *D. melanogaster* Y-genes (excluding Mst77y) have at least one convincing paralog (Table 1). The function of Y-genes and their paralogs are not known to be immunity-related. We propose the paralogs of Pp1-Y1, kl-5, kl-3, Ppr-Y, FDY, and CCY may also have immunity-related functions in flies during yeast and bacterial infections. Further studies need to be completed to determine how these genes contribute to flies’ immunity. Such studies may elucidate the function(s) and the evolution of *D. melanogaster* Y-genes.

Low conservation across Y-chromosomes exists across *Drosophila* species. Most of the *D. melanogaster* Y-genes are conserved in other *Drosophila* species but not necessarily on the Y chromosome [9]. For example, in *D. persimilis* and *D. pseudoobscura*, all Y-genes are encoded by the autosome [26]. Whether located on the Y chromosome or the autosome, we predict Y-genes of other *Drosophila* species to have a similar impact on survival to microbial infections as we observed here. If true, in species where Y-genes are encoded by the autosomal chromosomes, their effect on survival may no longer be male-specific. Furthermore, the effect of FDY in male sensitivity to *S. liquefaciens* should not be seen in other *Drosophila* species since FDY is exclusive to *D. melanogaster* [27].

**Figure 3:**
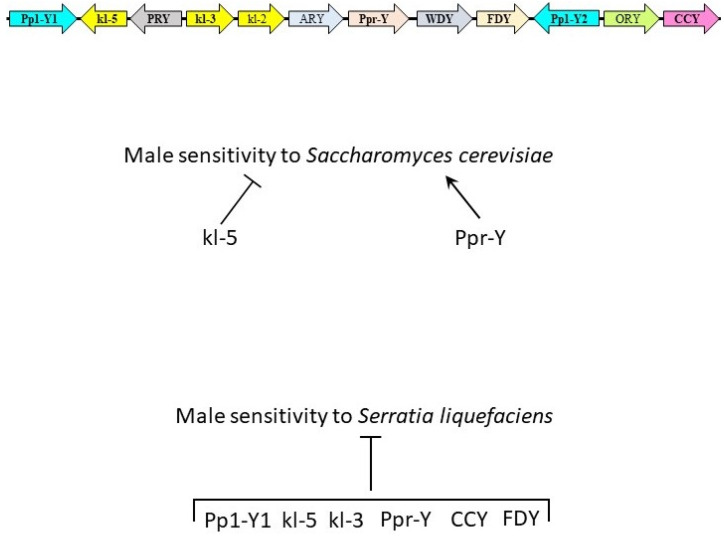
Genes of *Drosophila melanogaster* Y chromosome and their role in immunity. The Y chromosome of *D. melanogaster* consists of 12 single-copy protein-encoding genes. Loss of function of genes in bold were studied during fungal and bacterial oral infection assays. During *Saccharomyces cerevisiae* challenge, kl-5 and Ppr-Y decrease and increase the sensitivity of male flies to infection, respectively. Pp1-Y1, kl-5, kl-3, Ppr-Y, CCY, and FDY were found to decrease male sensitivity to *Serratia liquefaciens* oral infection.

Human orthologs of *D. melanogaster* Y-genes appear to be housekeeping genes (Table 1). While some human orthologs have very similar functions to fly Y-genes (protein phosphatases and dynein heavy chains), others possess more human-specific functions or are associated with human diseases (SERBP1 and CFAP58). All orthologs, except for DRC3 (a homolog for fly Ppr-Y), express mRNA in most major human tissues, where each gene is uniquely overexpressed in specific tissues [28]. PPP1CA, DNAH17, DNAH5, DNAH8, SERBP1, and CTTNBP2 proteins are overexpressed in lymph nodes, CD8+ T cells, B-lymphocytes, neutrophils, peripheral blood mononuclear cells, and monocytes, respectively. This further supports the role of these genes and their Y-gene homologs’ role in host immunity (Figure 3).

Previously, the function of many of Y-genes was only known to be fertility-related. Pp1-Y1, Pp1-Y2, and Ppr-Y belong to the phosphoprotein phosphatase (PPP) family, evolutionarily conserved enzymes responsible for dephosphorylating the serine and threonine protein residues [26]. Ppr-Y is similar to a dynein light chain component of *Chlamydomonas* flagella [29]. PRY has domain homology to polycystins linked to sperm storage and fertilization [14]. Disruption in kl-2, kl-3, and kl-5 results in loss of outer dynein arm of sperm tail axoneme, essential for male fertility [10]. WDY contains three WD40 domains, important for various functions, including adaptor/regulatory modules in signal transduction, pre-mRNA processing, and cytoskeleton assembly [14,15]. ORY is similar to occludin, a membrane protein in mice [29]. Other than their fertility role, the functions of PRY, ARY, FDY, and CCY are unknown [23]. Our study shows that the disruption of any of the Pp1-Y1, kl-5, kl-3, Ppr-Y, FDY, and CCY genes lead to the increased sensitivity of males to bacterial infection, which suggests that the protein products of these genes are members of the same pathway. More studies are needed to determine whether these Y-genes form an immunity-related pathway.

It has previously been shown that XXY mutant females do not express Y-genes [16,30]. Although the presence of a polymorphic Y-chromosome alone can increase the expression of several AMP genes within XXY females [16], there does not appear to be varying immune gene expression or functional immune response to bacterial infections of XXY females carrying Y-chromosomes from a single population [17]. Interestingly, males with varying Y-chromosomes do express significant immune response, which is significantly correlated to increased survival time to systemic *Serratia* infection. Here, we observed the presence of Y-chromosome not to affect XXY female survival to microbial infection. This may be due to the lack of expression of Y-genes in our XXY females or due to our use of oral infection where *Drosophila* gut immunity is primarily activated.

Natural oral infections of *S. cerevisiae* can be fatal for *Drosophila* and other insects and are used as a biopesticide in agriculture [31,32]. Pathogenic infections of yeast are dependent on fly fitness, as *S. cerevisiae* is also a commensal microorganism to *Drosophila*. To further enhance yeast effectiveness towards fruit flies, it could be possible to inhibit kl-5 to increase male susceptibility by introducing kl-5 inhibitors. Since kl-5 and Ppr-Y do not share close homology, kl-5 inhibitors should not inhibit Ppr-Y. Similarly, introducing loss of function kl-5 mutants into the wild population may achieve a similar effect. Additionally, fly mutants with loss of function in Pp1-Y1, kl-5, kl-3, Ppr-Y, CCY, and FDY can also be introduced into agricultural crop populations whose microbiota includes *S. liquefaciens* [33,34]. Male progeny of such fly mutants should also inherit the loss of function in the respective Y-gene and thus be more susceptible to entomopathogenic microbes.

## 5. Conclusions

This study hypothesizes that there are Y-genes that affect male sensitivity to microbes. While previous studies showed that the polymorphisms within the Y chromosome were sufficient to affect the host sensitivity, this study investigates the necessity of individual Y-genes for male sensitivity to microbes. We identified several Y genes whose genetic inactivation either increases or decreases the sensitivity of males to gastrointestinal fungal and bacterial infections. Moreover, the addition of the Y chromosome to females was not sufficient for the sexual dimorphism in the sensitivity to oral microbial infections. This study identifies the immune-related function of Y genes in *D. melanogaster*.

## Figures and Tables

**Table 1:** Paralogs and orthologs of *Drosophila melanogaster* Y chromosome genes. For fly and human genes, the percentage of identities and similarity to Y-genes is shown, as well as gene function.

*D. melanogaster* Y-Genes	*D. melanogaster* Paralog(s)		*H. sapiens* Ortholog(s)	
	Gene Name(s) (Identity %/Similarity %)	Gene Function(s)	Gene Name(s) (Identity %/Similarity %)	Gene Function(s)
Pp1-Y1: Protein phosphatase 1, Y-linked 1	Pp1-Y2 [63/75] flapwing [60/76] Pp1α-96A [60/75] Pp1-13C [59/76] Pp1-87B [59/75]	Protein serine/threonine phosphatase activity	Protein Phosphatase 1 Catalytic Subunits α, β, γ PPP1CA [57/74] PPP1CB [59/74] PPP1CC [59/75]	Serine/threonine protein phosphatase; glycogen metabolism, muscle contractibility, protein synthesis
kl-5: male fertility factor kl5	Dynein heavy chain Dhc93AB [61/76]	Ciliary and flagellar motor proteins	Dynein Axonemal Heavy Chain 9 DNAH9 [58/74]	Force generating protein of respiratory cilia
	CG3339 [55/71]	Unknown	Dynein Axonemal Heavy Chain 17 DNAH17 [59/75]	Force generating protein in sperm flagellum
PRY: polycystine-related-Y	CG30048 [26/44]	Unknown	Polycystin 1 Like 3, Transient Receptor Potential Channel Interacting PKD1L3 [20/36]	Calcium channel component
	CG42685 [24/42]	Predicted calcium channel activity		
kl-3: male fertility factor kl3	CG9492 [52/69]	Unknown	Dynein Axonemal Heavy Chain 5 DNAH5 [51/69]	Force generating protein of respiratory cilia
	Dynein heavy chain Dhc93AB [32/52] Dhc36C [32/51]	Ciliary and flagellar motor proteins	Dynein Axonemal Heavy Chain 8 DNAH8 [50/68]	Force generating protein in sperm flagellum
kl-2: male fertility factor kl2	Dynein heavy chain Dhc36C [35/54] Dhc16F [35/55]	Ciliary and flagellar motor proteins	Dynein Axonemal Heavy Chain 2 DNAH5 [33/53]	Force generating protein of respiratory cilia and in sperm flagellum
ARY: Aldehyde reductase Y	CG10638 [49/67]	Unknown	Aldo-Keto Reductase Family 1 Member B10 AKR1B10 [40/61]	Catalyzes Nicotinamide adenine dinucleotide phosphate NADPH-dependent reduction
	Akr1B [40/59]	Aldo-keto reductase 1B		
PPr-Y	TbCMF46 [61/80]	Unknown	Dynein Regulatory Complex Subunit 3 DRC3 [31/51]	Key regulator of ciliary and flagellar motility
WDY WD40 Y	CG34164 [60/73]	Unknown	EF-Hand Calcium Binding Domain 8 EFCAB8 [23/39]	Calcium ion binding
FDY: flagrante delicto	vig2 [93/94]	heterochromatin organization, histone H3-K9 methylation and chromatin silencing regulation	Serpin Family E Member 1 SERBP1 [44/62]	inhibitor of fibrinolysis
			Hyaluronan Binding Protein 4 HABP4 [33/50]	Regulates transcription, pre-mRNA splicing and mRNA translation
Pp1-Y2: Protein phosphatase 1, Y-linked 2	Pp1α-96A [75/88] Flw [76/89] Pp1-87B [74/87] Pp1-13C [73/86] Pp1-Y1 [63/75]	Protein serine/threonine phosphatase activity	Protein Phosphatase 1 Catalytic Subunits α, β, γ PPP1CA [57/74] PPP1CB [59/74] PPP1CC [59/75]	Serine/threonine protein phosphatase; glycogen metabolism, muscle contractility, protein synthesis
ORY: Occludin-Related Y	CG6059 [34/58] CG5882 [26/51]	Unknown	Cilia and Flagella Associated Protein 58 CFAP58 [29/54]	Associated with melanoma
CCY: Coiled-Coils Y	CG31161 [52/71]	Unknown	Cortactin-binding protein 2 isoform X7 CTTNBP2 [24/48]	Interacts with a central regulator of the actin cytoskeleton

## Data Availability

Data sharing not applicable.
